# Azithromycin concentrations during long-term regimen, a pilot study in patients with MALT lymphoma

**DOI:** 10.1038/s41598-021-97836-w

**Published:** 2021-09-16

**Authors:** Raphael Scheibenpflug, Markus Obermüller, Gottfried Reznicek, Ortrun Neuper, Wolfgang W. Lamm, Markus Raderer, Heimo Lagler

**Affiliations:** 1 Division of Infectious Diseases and Tropical Medicine, Department of Medicine I, Medical University of Vienna, Währinger Gürtel 18 – 20, 1090 Vienna, Austria; 2 Department of Pharmacognosy, University of Vienna, Vienna, Austria; 3 Division of Oncology, Department of Medicine I, Medical University of Vienna, Vienna, Austria

**Keywords:** Pharmacokinetics, Chemotherapy, Drug safety

## Abstract

In view of the antineoplastic effects of the macrolide clarithromycin in mucosa associated lymphatic tissue (MALT)-lymphoma, we performed a pilot study assessing levels of azithromycin in plasma, peripheral blood mononuclear cells (PBMC) and polymorphonuclear leukocytes (PMN) of MALT-lymphoma patients to determine the pharmacokinetics and potential influences of respective concentrations on the therapeutic outcome. In total 16 patients with MALT-lymphoma received 1.5 g of oral azithromycin once-weekly over 6 months. Blood was sampled directly prior to the following dose every 4 weeks during treatment. Drug levels were analysed by high performance liquid chromatography in plasma and intracellularly in PBMC and PMN. They were correlated with patients’ age, weight and body-mass-index and compared between patients responsive or unresponsive to treatment. Mean azithromycin plasma levels of all patients were 58.97 ± 30.48 ng/ml, remaining stable throughout the treatment period. Correlation analysis of plasma azithromycin showed no significance. Intracellular PBMC concentrations were 6648 ± 8479 ng/ml, without any significant difference between responders and non-responders. Mean PMN levels were 39,274 ± 25,659 ng/ml and significantly higher in patients unresponsive to treatment (t = 2.858, *p* = 0.017). Our drug regime led to continuously high plasma and exceedingly high intracellular concentrations of azithromycin in PBMC and PMN. Age, weight or body-mass-index had no significant influence on plasma levels and thence should not be considered in dosage finding. High AZM levels in PBMC did not lead to a better treatment response, whereas enrichment in PMN suggested a poorer outcome. The threshold for immunomodulatory effects on lymphoma cells might not have been reached. Additionally, the finding of stable plasma and intracellular concentrations over months with high-dose azithromycin administered in intervals might also be important for the further design of azithromycin-based trials against MALT-lymphoma.

Trial registration: EudraCT 2016-001521-13, 14/06/2016.

## Introduction

Lymphomas of the mucosa associated lymphatic tissue (MALT) develop in the background of inflammatory processes leading to the formation of extranodal lymphatic tissue at various mucosal sites^1^. Since the discovery of *Helicobacter pylori* as one of the causative agents for the development of gastric MALT lymphoma in 1991 and the following reports of lymphoma regression after antimicrobial *H. pylori* eradication, research has been done to identify the role of infectious agents and antimicrobial therapy in the pathogenesis and treatment of this disease^2–4^. Although radiotherapy, chemotherapy and monoclonal anti-CD20-antibodies offer excellent disease control, the indolent course, slow progression rate and the tendency towards dissemination of MALT lymphomas make them eligible for less aggressive therapies including antimicrobial approaches^5^. The connection between *H. pylori* induced chronic gastritis and gastric MALT lymphoma has been generally acknowledged and antimicrobial eradication therapy leads to remission in > 70% of cases^4,6,7^. Similarly, antimicrobial treatment of extragastric MALT lymphoma with doxycycline or clarithromycin has given promising results, with reported overall response rates of around 50%^4,7–11^. The potent therapeutic effects of the macrolide clarithromycin on MALT lymphomas are probably due to a combination of antimicrobial activity against causative bacteria and immunomodulatory properties^4,12^. Macrolides are known to reduce cytokine and nitric oxide secretion in leukocytes, decrease production of nuclear transcription factors and promote inflammatory cell apoptosis^13–19^. The macrolide azithromycin (AZM) has become of interest as a potential therapeutic option for treating MALT lymphomas due to its promising pharmacokinetic (PK) features and its superior immunomodulatory effects in vitro compared to clarithromycin^20,21^. Furthermore, AZM is known for its exceedingly high tissue penetration with even higher concentrations at inflammatory sites, macrophages and leukocytes (more than 100-fold serum concentration) and its long half-life of approximate 79 h^20^. AZM and clarithromycin have been shown to suppress T-cell activation and subsequent proliferation via interaction with the mTOR pathway. Furthermore, a reduction of T-cell cytokine secretion and induction of apoptosis in CD4 + cells has been shown. These effects were dose-dependent and AZM proved to be more potent, as four times higher concentrations of clarithromycin were needed to achieve similar effects^21^.

In view of these facts, AZM was selected for a phase II trial in MALT lymphoma patients^22^. This was also based on the fact that long‐term regimens of oral AZM had already been studied as antimicrobial and anti-inflammatory treatment of patients with cystic fibrosis and had been shown to be safe and well tolerated^23^.

### Objectives

The aim of this pilot study was to examine the concentrations of AZM in blood plasma, peripheral blood mononuclear cells (PBMC) and polymorphonuclear leukocytes (PMN) in patients with MALT lymphoma being treated with this long‐term, once‐weekly regimen of oral AZM over a maximum of 6 months^22^. We have hypothesized an association between the plasma and intracellular levels of AZM and the response to treatment as well as potential influence of age, weight or body mass index (BMI) on these respective concentrations.

## Patients and methods

### Blood sampling

All blood samples for this study were obtained from 16 patients (all > 18 years of age with a histologically verified MALT lymphoma accordant to the WHO Classification^24^ participating in a recent clinical study of oral azithromycin (MALT-A) performed at our centre^24^. Patient characteristics are shown in Table 1.

**Table 1:** Patient characteristics and location of the disease.

Variables	Total (n = 16)
Age	
Mean (range)	66.5 (47–88)
Median	68
Sex	
Male	7 (44%)
Female	9 (56%)
State of lymphoma	
Single organ involvement	9 (56%)
Multiple organ involvement	7 (44%)
MALT location	
Orbit	9
Stomach	2
Lung	2
Breast	2
Glandula parotis	1
Subcutaneous	1
Adrenal gland	1
Lymph node involvement	5 (31%)
ECOG status 0–1	100%

The extended MALT-A protocol, including blood sampling for further AZM plasma level measuring, had been approved by the Ethical Board of the Medical University of Vienna (approval no. 1383/2016) and performed in accordance with the Declaration of Helsinki (1964), Good Clinical Practice guidelines of the European Commission and the Good Scientific Practice guidelines of the Medical University of Vienna. Additionally, the MALT-A study was registered at EudraCT (approval no. 2016-001521-13, 14/06/2016) before initiation and written informed consent was obtained from all participants of this study. All methods were carried out in accordance with the relevant guidelines and regulation. AZM (Zithromax 500 mg tablets, Pfizer) was administered orally at a dose of 1500 mg once-weekly for 24 weeks. As a part of the protocol, for quantification of AZM trough levels in plasma and white blood cells 18 mL of blood were collected in tubes containing ethylenediaminetetraacetic acid (EDTA). The timepoints for blood-sampling were directly before the start of treatment (zero value) and on days 28, 56, 84, 112, 140 and 168, i.e. every 4 weeks. Blood was drawn right before the intake of the subsequent AZM administration^24^.

### Blood preparation

For assessment of AZM plasma levels, 9 ml EDTA blood were centrifuged at 500G (1800 rpm) for 10 min at room temperature and supernatant plasma was removed by pipette. For the separation of PMN and PBMC from 9 mL whole EDTA blood the ready-made separation system Polymorphprep (Axis-Shield, Oslo, Norway) was used according to the manufacturer’s instructions. The separated PMN and PBMC were then resuspended in 3 mL PBS and cell counts were performed by automated haematology analyser XN-30 (Sysmex, Kobe, Japan). Collected plasma and cell samples were continuously stored at -20 °C until final processing for measurement of AZM levels.

### AZM level measurement

For the measurement of plasma and intracellular AZM concentrations, a high performance liquid chromatography (HPLC)/triple quad mass spectrometry (MS) method was used ensuring selective and sensitive quantification of AZM levels^25–27^. The HPLC/MS parameters were optimized for the detection of AZM and the internal standard erythromycin (ERM). The following sample preparation procedure was used: 100 µl of plasma or cell samples was mixed with 100 µl of a solution of the internal standard ERM and 600 µl of methanol were added, mixed and centrifuged at 15.000 rpm after 10 min., the supernatant (5 µl) was analysed by LC/MS. AZM was quantified using HPLC–MS/MS on an Ultimate 3000 RSLC-series system (Dionex, Germering, Germany) coupled to a triple quadrupole mass spectrometer (AB Sciex Instruments API 4000) equipped with an orthogonal ESI source operated in positive mode^25–27^.

LC separation was performed on an Acclaim RSLC 120 C8 column (3 µm, 150 × 2.1 mm I.D., Thermo Fisher Scientific), preceded by an Acclaim 120 C8 guard cartridge (5 µm, 10 × 2 mm I.D., Thermo Fisher Scientific), at a flow rate of 0.5 mL/min and a column temperature of 25 °C. The mobile phase consisted of 50 mM aqueous ammonium acetate (mobile phase A), and methanol + acetonitrile (3 + 7) with 1% formic acid (mobile phase B). The analysis was carried out in isocratic mode with 44% B for 6.0 min, then purging with 95% B for 2.0 min, then again 44% B to equilibrate the column for 5.0 min before application of the next sample (total analysis time 13.0 min), AZM eluted at 2.42 min, ERM at 3.13 min. To avoid carry over a special user defined wash program was developed for the autosampler (washing solution methanol + acetonitril + 0.05 M aqueous ammonium acetate, 2 + 3 + 5). The autosampler tray was cooled to 5 °C. Selective and sensitive detection and quantification was carried out using MS/MS fragmentation of AZM giving a quasi-molecular ion at m/z 749.6 [M + H]^+^. MRM m/z 749.6/591.5 was used for calibration and quantification with the internal standard ERM (m/z 734.6 [M + H]^+^, MRM m/z 734.6/576.2), injection volume was 5 µl each. A linear concentration range was found from 5 to 1000 ng/mL (correlation coefficient 0.9996).

The triple quadrupole mass spectrometer was operated with the following parameters: ESI pos., IS + 4500, EP 10.0, CUR 10.0, GS1 40.0, GS2 40.0, TEM 500 °C, CAD 4.0, CEM 2500.0, DF -100.0, AZM: DP 96.0, CE 41.0, CXP 18.0, dwell 150 ms, ERM: DP 76.0, CE 31.0, CXP 32.0, dwell 150 ms.

For calculation of the intracellular concentrations a intracellular volume of 421 fL for PBMC and 334 fL for PMN was used, according to literature^28^.

### Statistics

The current study was a follow-up pilot study of a phase II clinical trial on the effects of AZM in MALT lymphoma patients^22^. The study design was that of a two-phase study: in phase 1 AZM is tested in 16 patients. The optimal sample size to test the null hypothesis (*p* < 0.05) would be 24.52. The risk of a type I error is 0.049, for a type II error 0.199. If 7 patients or less react to the drug, the study is cancelled. If phase II is reached, the drug is being tested on 46 patients. If less than 23 patients react, the drug is rejected.

For statistical calculations SPSS Statistics (IBM, Version 23) was used. All parameters were non parametrically tested using Spearman's rank correlation coefficient*.* For comparison of intracellular AZM levels between response groups the Mann–Whitney U test was used. For an analysis of inter- vs intrasubject variability the coefficient of variation $\left( {CV = \frac{\sigma}{\mu}} \right)$ was calculated. For calculation of the area under the concentration–time curve from dosing (time 0) to the last dose (AUC_0-last_) linear trapezoidal method was used.

## Results

A total of 16 patients (6 male, 10 female) with a mean age of 66.5 years (range 47–88 years) a mean weight of 76.9 kg (range 64–86 kg) and a mean height of 164 cm (range 145–189 cm) were recruited between August 2016 and October 2017 to receive a long-term, once-weekly regimen of oral AZM. For all 16 patients, at least one valid AZM plasma concentration could be determined, whereas concentrations in PBMC and PMN could be measured in 14/16 and 15/16 patients, respectively.

**Figure 1:**
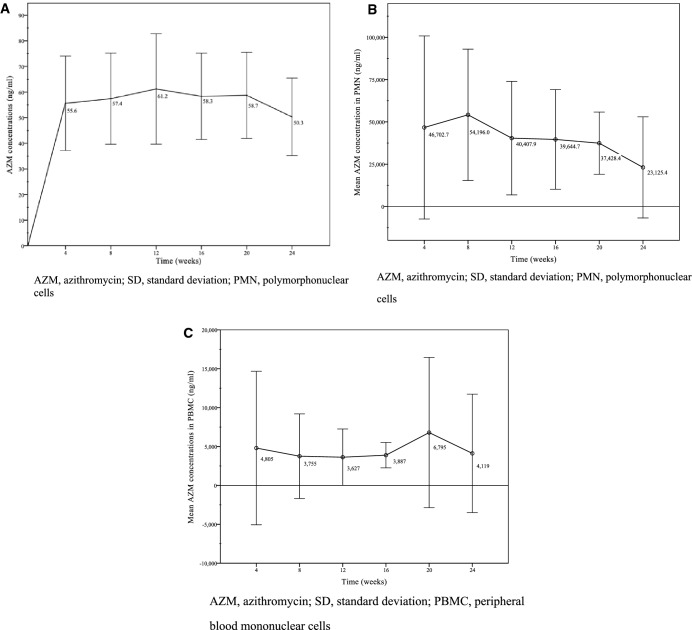
(**A**) AZM plasma trough levels (mean, ± SD; ng/ml) in MALT lymphoma patients (n = 15) at all given time points of AZM treatment over a period of 24 weeks. (**B**) Intracellular concentrations of AZM (mean, ± SD; ng/ml) in PMN of all patients (n = 16) over the period of treatment. (**C**) Intracellular concentrations of AZM (mean, ± SD; ng/ml) in PBMC of all patients (n = 16) over the period of treatment. *AZM* azithromycin, *SD* standard deviation, *PBMC* peripheral blood mononuclear cells.

The mean AZM plasma trough level of all patients at all given time points was 58.97 ng/ml (standard deviation (SD) ± 30.48 ng/ml). As shown in Fig. 1, AZM levels were relatively constant during the first 20 weeks and slightly decreased at week 24. Notably, there was one patient (no. 3) whose mean AZM levels were more than three standard deviations higher than the mean (150.07 ng/ml). Due to missing values AUC calculations were only done for AZM plasma concentrations. The mean plasma AUC_(0-last)_ was 160.29 h × µg/mL (SD ± 112.53 h × µg/mL). Summarized PK statistics are listed in Table 2.

**Table 2:** Summary statistics for pharmacokinetic parameters.

Statistic	c_plasma_ (ng/mL)	Plasma AUC_(0-last)_ (h × µg/mL)	c_PBMC_ (ng/mL)	c_PMN_ (ng/mL)
N	16	14	14	14
Mean	58.97	160.28	6647	39,274
SD	30.48	112.53	8479	25,659
Min	22.42	76.24	593	7231
Median	54.05	169.26	2911	39,368
Max	80.91	476.85	30,957	94,875

Before running correlation analysis, the variables of mean AZM concentrations (ng/ml) at all time points, age (years), body weight and calculated BMI (kg/m^2^) were checked for normal distribution. All variables but patients’ age were normally distributed, due to the small sample size we decided to use *Spearman's rank correlation coefficient.* We found no significant correlation between these variables. The results are listed in Table 3.

**Table 3:** Correlation analysis for patient’s age (years), body-mass-index (BMI) and mean azithromycin (AZM) plasma concentration (ng/ml) at all time points using Spearman’s rank coefficient for non-parametric testing.

	N	Median	IQR	Spearman-Rho	*P*
Age (years)	16	68.0	18	0.462	0.084
BMI (kg/m^2^)	14	28.3	16.3	0.126	0.681
Weight (kg)	16	76.5	9.1	-0.168	0.533

**Figure 2:**
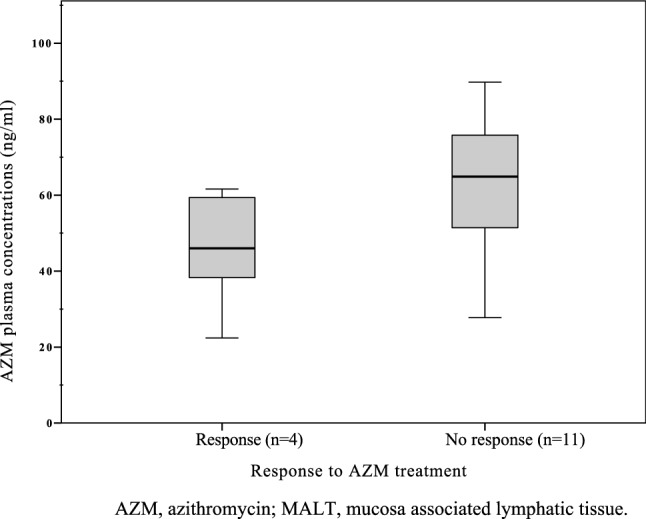
AZM plasma trough levels (mean, ng/ml) in MALT lymphoma patients (n = 15) with response or no response to AZM treatment over a period of 24 weeks. *AZM* azithromycin, *MALT* mucosa associated lymphatic tissue.

To detect a possible association of AZM plasma concentrations and response to treatment, we divided patients in two groups according to response of the lymphoma to the AZM therapy. The therapeutic results have already been published^22^. Patients with stable disease (StD) or progressive disease (PD) during the study were rated as unresponsive to treatment (non-responder), those having partial remission (PR) or complete remission (CR) of the disease as responsive to treatment (responder). As reported by our MALT lymphoma study group 4/16 patients (25%) responded (2 PR, 2 CR), 9 patients (56%) had StD and 3 patients were considered PD (19%)^22^. We compared the means of both groups using a non-parametric approach (Mann–Whitney U test). Though the mean AZM plasma trough levels in the unresponsive group seemed higher (Fig. 2), no significant difference was found between responders versus non-responders, which might have been due to the small number of patients especially in the responder-cohort.

**Figure 3:**
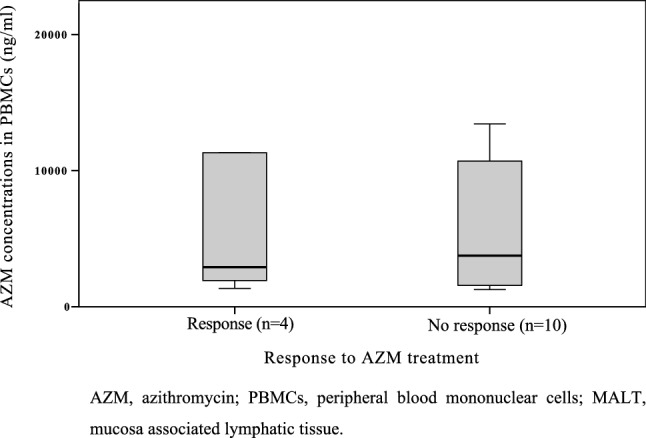
Intracellular levels of AZM (mean, ng/ml) in PBMCs in MALT lymphoma patients (n = 14) with response or no response to AZM treatment over a period of 24 weeks. *AZM* azithromycin, *PBMCs* peripheral blood mononuclear cells, *MALT* mucosa associated lymphatic tissue.

Assessment of PBMCs showed an overall mean intracellular AZM concentration of 6648 ng/ml (SD ± 8479 ng/ml) (Fig. 1C). The data were heterogeneously distributed and did not fit the criteria for parametric testing between the two response groups (Fig. 3); application of Mann–Whitney *U* testing showed no significant difference (*p* = 1.00).

**Figure 4:**
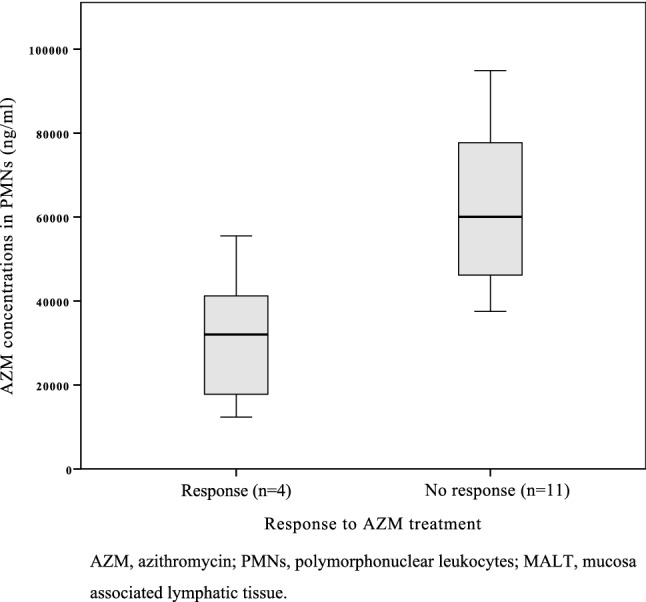
Intracellular levels of AZM (mean, ng/ml) in PMNs in MALT lymphoma patients with response or no response to AZM treatment over a period of 24 weeks. *AZM* azithromycin, *PMNs* polymorphonuclear leukocytes, *MALT* mucosa associated lymphatic tissue.

The overall mean intracellular AZM concentrations in PMNs were 39,274 ng/ml (SD ± 25,659 ng/ml) (Fig. 1B). As shown in Fig. 4, the median AZM levels in the 12 patients unresponsive to therapy were higher. The mean AZM concentrations in this group were 62,728 ng/ml (SD ± 21,303 ng/ml), whereas those patients responding to therapy reached mean levels of 31,822 ng/ml (SD ± 15,745 ng/ml). The groups were thus compared using Mann–Whitney U test, which showed a significant difference (*p* = 0.026).

## Discussion

To our knowledge this pilot study is the first report of plasma and intracellular levels (in PMN und PBMC) of AZM in MALT lymphoma patients undergoing continuous oral treatment for 6 months. We found the mean plasma concentrations (58.97 ng/ml, SD ± 30.48) observed in our patients to be relatively high and stable over the whole period of application (Fig. 1A). Yet at week 24 AZM levels appeared to be slightly decreased. However, this fact should be interpreted with caution as we lost follow-up of 6 patients at week 24 and plasma levels used to be above average in these patients.

A study by Amsden et al. was the only one using 1500 mg AZM as a single shot therapy in healthy volunteers^29^. After 7 days they reported mean plasma levels of about 30–40 ng/ml. Other study groups used different dosage regimes: Matzneller et al. applied AZM 500 mg for 3 days and plasma concentrations determined 5 and 10 days after the beginning of treatment were 27.8 and 8.15 ng/ml^30^. Olsen et al. used 500 mg once, followed by 4 daily doses of 250 mg AZM. 7 days after the last dose they reported plasma concentrations of 15 ng/ml^31^.

In accordance with these previous studies on healthy individuals^29–31^, we conclude that a dosage regime of 1500 mg AZM once weekly in patients with MALT-lymphoma provides continuously high blood levels and, as AZM is distributed quickly from plasma to various tissues and leukocytes with considerable accumulation, correspondingly high concentrations in these compartments.

In order optimize future treatment approaches, we were interested in potential influences on azithromycin distribution in blood plasma, such as age, body weight and BMI. Due to its rapid redistribution from the vascular compartment to tissues and leukocytes, the plasma concentrations of AZM after several days are mainly a result of slow efflux from these compartments rather than biliary or renal elimination. Thus, it can be suspected that individual distributional differences of adipose, muscular and lymphatic tissue as well the extent of local inflammation with associated cell accumulation are the main constituents of AZM plasma concentrations and that these parameters are not sufficiently represented by measuring weight and BMI alone.

One of our patients has shown particularly high plasma through levels which were more than three standard deviations above the mean. In fact, this patient had the lowest body weight with 41.0 kg, which might explain the alteration. Still, we were unable to find any significant correlation between body weight and AZM plasma concentrations (Table 1).

Aging is typically accompanied by an increase of fat tissue, a relative reduction in muscle mass, as well as a decrease in glomerular filtration rate^32^. In our patients with an age ranging from 47 to 88 years and a mean age of 66.5 years these influences might be of particular relevance.

However, in respect of clinical implications we found plasma concentrations to be independent of the factors we examined and therefore an adaptation of AZM dosing seems unnecessary. Of course, one has to take into account that one limitation of our study was the rather small study population and therefore there might have been a lack of statistical power to expose clinically relevant differences. We are aware, that the concentrations we measured in plasma and intracellularly show considerable variance. This might be explained by the uncertainty of measurement which would expected to be more prominent when working with very low concentrations. Assessing AZM levels by HPLC methods, however, has proven to be reliable even at very low concentrations^33^. Taking a closer look at the variation of AZM intracellular concentrations we compared inter- versus intrasubject variability using the coefficient of variation (CV). In both PMN und PBMC intracellular concentrations varied more prominently in between subjects (CV_PMN_ 1.20; CV_PBMC_ 1.28) than within subjects (CV_PMN_ 0.64; CV_PBMC_ 0.72). This supports the solidity of the intracellular measurements. However, complex kinetics of AZM accumulation along with a varying state of inflammation (as discussed below) might be an explanation for this.

Regarding intracellular levels of AZM, we measured higher drug levels in PMN than in PBMC (39,274 vs. 6648 ng/ml) (Figs. 3, 4). These results are not in line with those of Amsden et al., who measured higher concentrations in PBMC than PMN (27 vs. 18 mg/l) 10 days after application of 1500 mg AZM^29^. Wildfeuer et al., on the other hand, reported 53 mg/l in PMN and 1 mg/l in PBMC 14 days after an administration of 500 mg AZM for 3 consecutive days. However, none of those patients had undergone continuous dosing, and different methods in the handling of the blood samples could also be an explanation. In fact, Wildfeuer et al. described losses of about 25% of AZM from monocytes during one step of washing with PBS. Furthermore, when calculating intracellular concentrations, different study groups used different cell volumes (e.g. Wildfeuer et al. for PMN 450 fl; Amsden et al. for PMN 334 fl)^34^. That indicates that the measured concentrations are not directly comparable with those of other study groups.

Furthermore, the reported studies were performed on healthy subjects whereas pathophysiological differences in lymphoma patients might contribute to the pattern of AZM distribution in this group. MALT lymphoma is characterized by chronic inflammation^35^. Due to azithromycin being a weak base it accumulates at sites of low pH, such as tissues with ongoing inflammation and intracellularly in lysosomes and phagosomes^36^. Our study population involved patients with MALT lymphomas of different origin, stage and disease activity. Taken into account that MALT lymphoma appears as a systemic disease in a multitude of cases, these factors might be, at least partly, an explanation for the heterogeneity of our data^37^.

Our expectation was, that as concentrations in PBMC might be similar to those of MALT lymphoma-lymphocytes, their determination could show a link between AZM dosing and treatment outcomes. Specifically, we were interested if failure to treatment might be due to insufficient AZM concentrations in lymphocytes. Our data did not hint at such a connection. However, in vitro studies by Ratzinger et al. show that relevant mTOR inhibitory concentrations require AZM concentrations of 10 mg/l in the culture medium, indicating far higher intracellular levels in lymphocytes under these conditions^21^. Achieving such levels in vivo reasonably and safely is improbable. Nevertheless, further studies on AZM effects on MALT lymphomas using higher doses might be promising. It has been shown that even high daily doses (15 mg/kg/day over 24 weeks) of AZM in patients treated for cystic fibrosis were well tolerated^38^. Apart from those data, we think that determining AZM concentrations in biopsy material of MALT lesions might be the most direct way to assess the situation on site. One could then evaluate, whether the local and the intracellular concentrations in PBMC and PMN are corresponding.

Interestingly, the AZM levels in PMN were significantly higher in patients without a response to treatment (Fig. 4). This result has to be taken with some caution, as we were only able to compare 4 patients responding to therapy with 11 non-responders.

In case of gastric MALT-lymphoma, it is known that the ongoing inflammation attracts and activates neutrophils. Patients who were unresponsive to AZM therapy had either stable or progressing disease, indicating a greater disease activity and level of inflammation in this subgroup. Due to the pharmacological properties of AZM mentioned before, one can suspect an accumulation of AZM in PMN based on the increased disease activity in these patients, which would explain why this patient subgroup suffered a poor outcome.

## Conclusion

We come to the conclusion that administering orally AZM high-dose once-weekly for several months results in continuously high plasma and correspondingly high intracellular concentrations in PBMC and PMN.

The distribution of AZM in MALT lymphoma patients appears to be unaffected by patients age, body weight and BMI and therefore we see no indication to adapt treatment dosage according to those factors. In our setting, we did not find proof that the response to treatment was in any way dose dependent, although intracellular levels might not have reached a sufficient magnitude for AZM to unfold immunomodulatory effects on lymphoma cells altogether.

The concentrations in polymorphonuclear leucocytes appeared to be significantly higher in patients who were unresponsive to treatment. This might be explained by increased disease activity and a higher level of inflammation in these patients.

Overall, we think that AZM shows promising effects in the treatment of MALT lymphoma and further clinical studies could benefit from higher AZM dosing. Additional measurements of AZM levels in biopsy material of MALT lesions as well as lager study populations will be necessary in the future.

## Data Availability

The data generated and analyzed in this study are available from the authors on request.
